# Prevalence of hypertension among type 2 diabetes mellitus patients in Ethiopia: a systematic review and meta-analysis

**DOI:** 10.1093/inthealth/ihac060

**Published:** 2022-09-02

**Authors:** Teklehaimanot Gereziher Haile, Teklewoini Mariye, Degena Bahrey Tadesse, Gebreamlak Gebremedhn Gebremeskel, Guesh Gebreayezgi Asefa, Tamirat Getachew

**Affiliations:** School of Nursing, College of Health Sciences, Axum University, Tigray, Ethiopia; Department of Adult Health Nursing, School of Nursing, Axum University, Tigray, Ethiopia; Department of Adult Health Nursing, School of Nursing, Axum University, Tigray, Ethiopia; Department of Adult Health Nursing, School of Nursing, Axum University, Tigray, Ethiopia; Department of Midwifery, College of Health Sciences, Axum University, Tigray, Ethiopia; School of Nursing and Midwifery, College of Health and Medical Science, Haramaya University, Ethiopia

**Keywords:** Ethiopia, hypertension, prevalence, type 2 diabetes mellitus

## Abstract

**Background:**

Hypertension among diabetic patients is a worldwide public health challenge and a leading modifiable risk factor for other cardiovascular diseases and death. This study aimed to estimate the prevalence of hypertension among diabetic patients in Ethiopia.

**Methods:**

The studies were selected using PubMed, Embase, Health InterNetwork Access to Research Initiative and Cochrane Library databases and Google searches. Two independent authors carried out the data extraction using a predetermined and structured method of data collection. R version 3.5.3 and RStudio version 1.2.5003 were used for analysing the data. To assess possible publication bias, funnel plot test methods were used. The guidelines of the Preferred Reporting Items for Systematic Reviews and Meta-Analyses were used to publish the results. This study was registered in the Prospective Register Systematic Reviews (CRD42020170649).

**Results:**

A total of 218 articles were identified but only 6 six full-text abstract papers were included in this systematic review and meta-analysis. The random effects model analysis showed that the pooled prevalence of hypertension among type 2 diabetes mellitus (DM) patients in Ethiopia was 55% (95% confidence interval [CI] 49 to 61). The subgroup analysis of the pooled prevalence of hypertension among type 2 DM patients in the Oromia and Southern regions was 51% (95% CI 42 to 59) and 58% (95% CI 54 to 63), respectively. The pooled prevalence of hypertension among type 2 DM patients was higher among urban residents (60% [95% CI 54 to 67] and 52% [95% CI 41 to 63] among urban and rural residents, respectively).

**Conclusions:**

This study showed a high pooled prevalence of hypertension among type 2 DM patients in Ethiopia. Appropriate preventive measures should be implemented to reduce the burden of hypertension among DM patients in Ethiopia.

## Introduction

Hypertension contributes enormously to the global disease burden and mortality. The prevalence of hypertension among type 2 diabetes mellitus (DM) patients is higher than that of age- and sex-matched patients without diabetes, ranging from 32% to 82%.^1^ It is a disorder in which the blood pressure (BP) is abnormally high and is described as systolic BP ≥140 mmHg and/or diastolic BP ≥90 mmHg.^2–4^

Globally, cardiovascular diseases account for approximately 17 million deaths per year, nearly one-third of the total of all worldwide deaths.^2^ Hypertension is one of the principal causes of global disease burden and is estimated to cause 7.5 million deaths, about 12.8% of all annual deaths throughout the world.^2–5^ According to the Global Health Observatory Report, the overall prevalence of hypertension in adults ≥25 y of age was around 40% in 2008.^6^

Hypertension among diabetic patients is a worldwide public health challenge and a leading modifiable risk factor for other cardiovascular diseases and death.^7^ The frequency of hypertension among the diabetic population is almost twice that of non-diabetic patients, as reported by previous epidemiologic studies.^8,9^ Also, compared with other cardiovascular disorders, hypertension is the most common comorbid disease in diabetic patients.^10^ According to 2009 WHO report, up to 80% of people with diabetes will die of cardiovascular disease,^5^ especially hypertension and stroke since most patients with diabetes develop hypertension. One of the key risk factors for cardiovascular disease is hypertension and it is present in all populations elsewhere in the world.^2,11,12^

Globally, the number of hypertensive adults in 2000 was estimated at 972 million: 333 million in economically developed countries and 639 million in economically developing countries.^13^ The number of adults with hypertension in 2025 is expected to increase to a total of 1.56 billion.^13^ Also, mortality increases 7.2 times in patients with diabetic hypertension.^13^ To tackle the burden of hypertension, the Pan-African Society of Cardiology identified 10 action points to be implemented by African ministers to achieve a 25% decrease by the end of 2025.^11^

Currently Ethiopia has been challenged by the increasing number of non-communicable diseases, including hypertension and DM.^14,15^ A meta-analysis conducted in Ethiopia revealed that the prevalence of type 2 DM was 6.5% and the prevalence of hypertension was 20.63%.^16^ The burden of DM and its cardiovascular complications like hypertension, peripheral neuropathy, nephropathy, coronary artery disease and stroke were currently increased in Ethiopia.^17,18^ Furthermore, early detection of hypertension and related cardiovascular risk factors is important to limit the complications of DM, so it is vital to determine the prevalence of hypertension in diabetic patients in Ethiopia.^17^

Although the pooled prevalence of hypertension in Ethiopia was reported previously,^19–21^ there is no national research on the prevalence of hypertension among DM patients in Ethiopia. Some regional studies have been conducted in different parts of Ethiopia and there is no pooled prevalence of hypertension among DM patients in Ethiopia. Thus this study aimed to estimate the pooled prevalence of hypertension at a national level using the findings obtained from these smaller regional studies.

## Methods

### Study protocol and systematic review registration

This meta-analysis and systematic review were conducted to identify the pooled prevalence of hypertension among DM patients in Ethiopia. The review was based on the Preferred Reporting Items for Systematic Reviews and Meta-Analyses (PRISMA) guideline^22^ (Supplementary file 1). This study is registered in the Prospective Register Systematic Reviews (PROSPERO) database (CRD42020170649).

### Study design and setting

Ethiopia covers an area of 1.1 km^2^, divided into nine regions (Tigray; Afar; Amara; Oromo; Somali; Benishangul-Gumuz; Southern Nations, Nationalities, and People's Region; Gambella and Harari) and two administrative states (Addis Ababa City and Dire-Dawa City). A systematic review and meta-analysis that included all articles that reported the prevalence of hypertension among type 2 DM patients in Ethiopia were conducted. All included articles were cross-sectional studies.

### Search strategy and data source

The studies were searched using the PubMed, Embase, Health InterNetwork Access to Research Initiative and Cochrane Library databases and previous prevalence lists from Google searches. The search was performed using keywords and Boolean operators (AND and OR) either individually or in combination using the following keywords: hypertension, Ethiopia, prevalence, blood pressure, systolic, diastolic, diabetic mellitus (Supplementary file 2).

### Data extraction and quality assessment

Two independent authors carried out the data extraction using a predetermined and structured method of data collection. The titles were independently reviewed by two authors (TGH and DBT), abstracts of all citations were obtained and the full-text search results found six qualifying studies. Data extraction included title, first author, publishing year, survey year, research type, research base (population and hospital-based), sample size, response rate and study area. Disagreements were settled through dialogue and consensus between the two authors.

### Criteria for considering studies for the review

Inclusion criteria were published observational studies from inception to 31 December 2020, type 2 diabetic patients, only peer-reviewed and published articles written in the English language, institutional and community-based studies with the prevalence of hypertension as the outcome. Exclusion criteria were studies that did not disclose the prevalence of hypertension among type 2 DM patients, experimental studies, reviews, commentaries and case series/reports.

### Quality assessment and risk of bias in individual studies

The methodological consistency of the studies included was assessed using the Hoy et al.^23^ tool for assessing the risk of bias in prevalence studies. The tool was designed to assess the quality of the meta-analysis of non-randomized studies. The tool contains 11 items: items 1–4 assess the external validity, 5–10 assess the internal validity and item 11 is a summary of the reviewer's overall risk assessment based on the responses of the above 10 items scored 0 if yes and 1 if no. Studies were rated as low risk (<3), moderate risk (4–6) or high risk (7–9) of bias. Two reviewers performed this exercise and disputes were resolved through dialogue and, where possible, through arbitration involving a third author. In addition, adequate sampling methods, consistent methods and procedures for collecting data and the representative sample size were considered indicators of the quality of the study. Studies of high quality were studies that revealed all the points mentioned above (Supplementary file 3).

### Data management

A framework was developed to guide the screening and selection process based on the inclusion and exclusion criteria. Before data extraction was started, the tool was piloted and revised. The search results were uploaded to Endnote version X8 software (Clarivate, Philadelphia, PA, USA) in order to delete duplicates.

### Data analysis and presentation of results

The characteristics of included studies (authors, publication, study year, study area/region, study design, study setting, sample size, number of cases, prevalence of hypertension among DM patients) were extracted and summarized in Excel 2016 (Microsoft, Redmond, WA, USA). The quantitative data were extracted from the included studies and stored in Excel 2016. Extracted data were exported to R version 3.5.3 (R Foundation for Statistical Computing, Vienna, Austria) and RStudio version 1.2.5003 (RStudio, Boston, MA, USA) for analysis. Forest plots were drawn to represent the aggregate prevalence of hypertension among type 2 DM patients and the degree of statistical heterogeneity between studies. The statistical heterogeneity was evaluated using the χ^2^ test and quantified using calculation of the I^2^ statistics, with values of 25%, 50% and 75% representative of low, medium and high heterogeneity, respectively.^24^ There was heterogeneity between studies. We therefore used a meta-analysis of random effects to estimate the aggregate pooled prevalence of hypertension among type 2 DM patients. To assess possible publication bias, funnel plot test methods were used.

### Selection and data collection process

Data were extracted using a standardized method of data extraction. The Joanna Briggs Institute Meta-Analysis of Statistics Assessment and Review Instrument adapted for cross-sectional/case–control study designs was used.^25^ Studies that were carried out in Ethiopia and reported the prevalence of hypertension were selected for the meta-analysis. After results were collected, two authors independently reviewed the names and abstracts of publications extracted from the reviewed articles against the inclusion criterion. As described above, disagreements were resolved by dialogue and consensus and another author (TG) arbitrated as necessary. Full texts for the qualified titles and/or abstracts and those where there was confusion were collected for further consideration as to whether they should be included in the study. Authors were contacted for additional details as necessary. Up to three e-mails were sent to the corresponding author to request additional information before excluding. Surveys that appear in one article with multiple surveys conducted at different time points were treated as separate studies.

### Data items

Data extraction included the first author, publishing year, country and/or region, sample size, type of publication, study area and characteristics of the study (study design, response rate).

### Outcomes and prioritization

The primary outcome was the prevalence of hypertension among type 2 DM patients in Ethiopia.

### Data synthesis

The original articles were represented using a forest plot and table. Because there was heterogeneity among the studies, a random effects model^26^ was used to determine the pooled prevalence of hypertension in Ethiopia. Geographic regions where the study was conducted were summarized by a subgroup analysis. Heterogeneity was investigated using Cochrane's Q, and I^2^ statistics were quantified.^24^ Results with corresponding 95% confidence intervals (CIs) were presented. The findings of this analysis were published based on the PRISMA guidelines.^22^

## Results

### Screening flow

**Figure 1.**
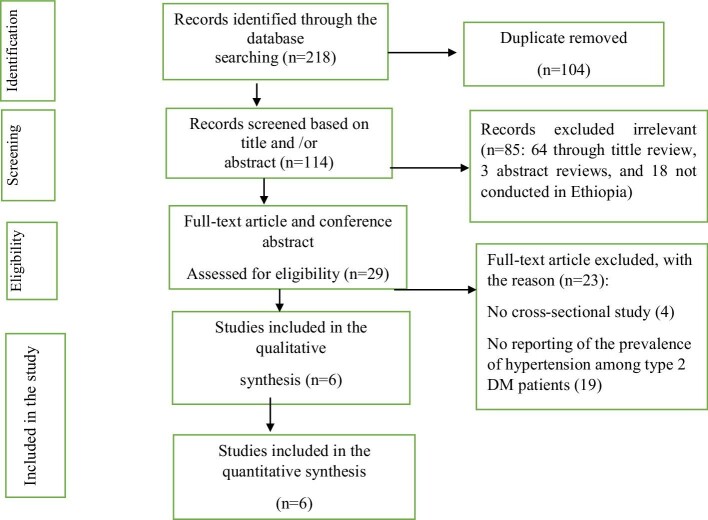
Selection of studies for a systemic review and meta-analysis of the prevalence of hypertension among type 2 DM patients in Ethiopia.

A total of 218 studies were identified from the databases and manual searches. From this, 104 of the studies were removed due to duplication. The remaining 114 studies were screened by their title and abstract and 85 of the studies were excluded (64 through title review, 3 abstract review and 18 studies not conducted in Ethiopia), while 23 studies were excluded due to not reporting the prevalence of hypertension among type 2 DM patients. Finally, a total of six studies fulfilled the inclusion criteria and were enrolled in this study (Figure 1).

### Study characteristics

This systematic review and meta-analysis included a total of six studies. About half (50%) of the studies were in the Oromia region, 33% were from the Southern region and 17% were from the Amhara region. The studies were all institutional-based cross-sectional studies (Table 1).

**Table 1.** Characteristics of studies included in the systematic review and meta-analysis of the prevalence of hypertension among type 2 DM patients in Ethiopia, 2020

Author	Publication year	Study area/region	Study design	Study setting	Sample size	Cases	Prevalence (%)
Dedefo et al.^18^	2018	Adama/Oromia	Cross-sectional	Institution based	382	215	56.30
Tadesse et al.^15^	2018	Hosanna/Southern	Cross-sectional	Institution based	140	77	55
Muleta et al.^43^	2017	Jimma/Oromia	Cross-sectional	Institution based	131	74	46
Kotiso et al.^17^	2020	Hosanna/Southern	Cross-sectional	Institution based	365	218	59.70
Dagnew and Yeshaw^28^	2019	Jimma/Oromia	Cross-sectional	Institution based	315	129	41
Akalu and Belsti^27^	2020	Debre Tabor/Amhara	Cross-sectional	Institution based	378	225	59.5

### The pooled prevalence of hypertension

**Figure 2.**
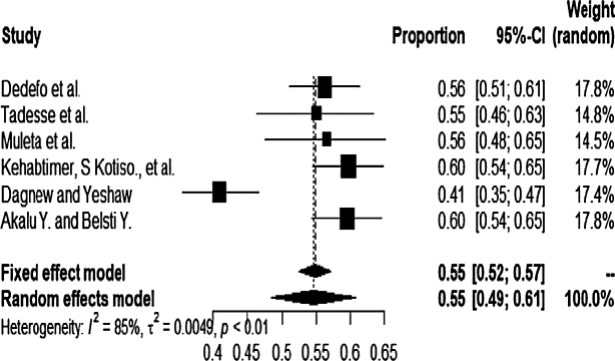
Forest plot of the six studies that assessed the pooled prevalence of hypertension among type 2 DM patients in Ethiopia, 2020.

In the analysis of six studies according to the random effects model, Der Simonian-Laird showed that the pooled prevalence of hypertension among type 2 DM patients in Ethiopia was 55% (95% CI 49 to 61). In this study, the heterogeneity was tested and I^2^=85% and p<0.001 indicated the presence of heterogeneity (Figure 2).

### Funnel test

**Figure 3.**
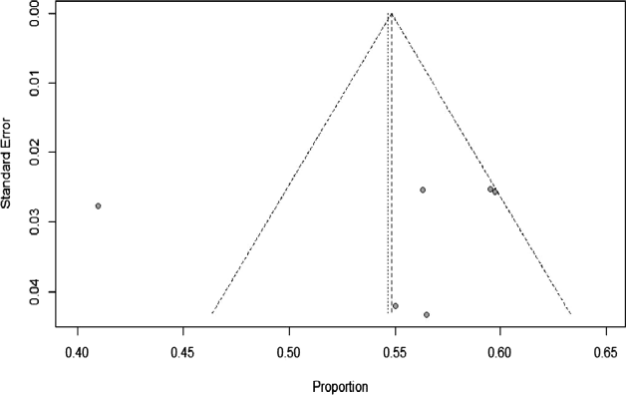
Funnel plot–indicated publication bias among studies.

Publication bias was assessed using the funnel test. The funnel plot found that the risk of bias for one article and the result according to Hoy et al.^23^ was moderate (6) (Figure 3).

### Subgroup analysis by region of the country

**Figure 4.**
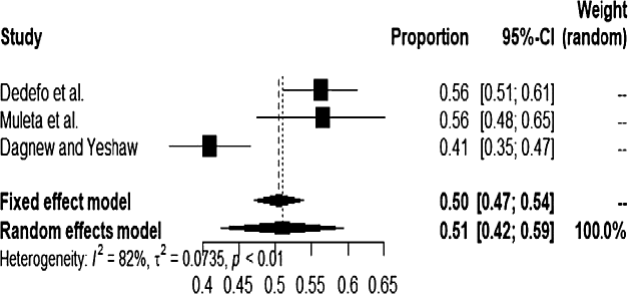
Forest plot for the subgroup analysis of the Oromia region.

**Figure 5.**
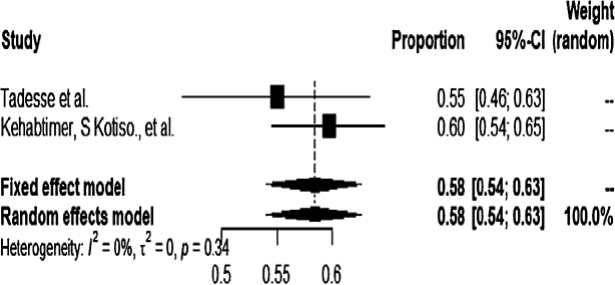
Forest plot for the subgroup analysis of the Southern region.

Most studies were carried out in the Oromia region. The pooled prevalence of hypertension among type 2 DM patients during the subgroup analysis in the Oromia and Southern regions was 51% (95% CI 42 to 59) and 58% (95% CI 54 to 63), respectively (Figure 4 and Figure 5).

### Subgroup analysis by gender and residence of primary study participants

The subgroup analysis shows that the pooled prevalence of hypertension among type 2 DM patients is comparable among males and females in Ethiopia (52% [95% CI 37 to 68] and 52% [95% CI 43 to 60], among males and females, respectively). On the other hand, the pooled prevalence of hypertension among type 2 DM patients during the subgroup analysis among urban and rural residents was 60% (95% CI 54 to 67) and 52% (95% CI 41 to 63), respectively (Table 2).

**Table 2.** Subgroup analysis by gender and residence of the primary study participants indicating the prevalence of hypertension among type 2 DM patients in Ethiopia

Characteristics	Studies included, n	Prevalence (95% CI)	Heterogeneity statistics	I^2^ (%)	p-Value	τ^2^
Gender						
Male	6	52 (37 to 68)	127.93	96.09	<0.001	0.04
Female	6	52 (43 to 60)	25.50	80.39	<0.001	0.01
Residence						
Urban	5	60 (54 to 67)	15.76	74.61	<0.001	0.00
Rural	5	52 (41 to 63)	20.67	80.65	<0.001	0.01

## Discussion

This systematic review and meta-analysis determined the pooled prevalence of hypertension among type 2 DM patients in Ethiopia using six studies. Based on the findings of this meta-analysis, the pooled prevalence of hypertension among type 2 DM patients in Ethiopia was 55%. As compared with the systematic review and meta-analysis done in Ethiopia that revealed only 19.6% of the general population had hypertension,^19,21^ the prevalence of hypertension among diabetic patients is almost three times higher than in non-diabetic populations. This implies that patients with chronic diseases like diabetes have a greater risk of becoming hypertensive patients.

The studies that were conducted in Hosanna, Southern Ethiopia and Debre Tabor, Northwest Ethiopia^15,27^ reported the highest prevalence of hypertension among type 2 DM patients (60%) in Ethiopia, while lower prevalence (41%) of hypertension among type 2 DM patients in Ethiopia was reported in a study done at Jimma University Specialized Hospital.^28^ This analysis highlights the major and urgent need to concentrate on the epidemiology of hypertension among type 2 DM patients in Ethiopia to fully understand the situation and undertake relevant action plans that will hopefully reduce morbidity and mortality due to hypertension and its related complications across the country.

The overall pooled prevalence of our findings is in line with the studies conducted in Saudi Arabia and Nigeria.^29,30^ Additionally, the studies carried out in other places such as southeast Nigeria, India and Oghara, Nigeria^31–33^ were consistent with this meta-analysis result. Our finding is high when we compared it with a study conducted in Bellary, India (25.6%).^34^ This discrepancy might be due to cultural and socio-economic differences. Moreover, the existence of publication bias in this review might conceal the true prevalence of hypertension among type 2 DM patients in Ethiopia. This type of bias commonly results in studies that have statistically significant positive results being published and statistically insignificant or negative studies not being published.^35^

The result of the subgroup analysis showed the prevalence of hypertension among type 2 DM patients is comparable among males and females in Ethiopia. This consistent result was also reported in a worldwide data analysis report regarding the global burden of hypertension.^13^ However, the prevalence of hypertension among type 2 DM patients is higher among urban residents. This is in line with systematic review and meta-analysis studies in Ethiopia,^19,21^ a study conducted in four sub-Saharan countries^36^ and a study conducted in Uganda.^37^ This higher prevalence of hypertension among type 2 DM patients in urban areas might be due to a sedentary lifestyle and changes in dietary habits following increasing urbanization. Decreased physical activity due to office work or the use of motorized transportation, consuming more processed foods and engaging in jobs with minimal physical activity could be possible reasons.

This study also revealed that the prevalence of hypertension among type 2 DM patients in the Southern region (58%) was slightly higher than in the Oromia region (51%). This variation may be due to the study year difference, variation in the management of DM and socio-economic factors of the study participants across the two regions.

The pooled prevalence of our findings is lower than that in studies conducted in Botswana, Jordan, India, Punjab, Libya, Israel and Iraq.^8,9^,^38–42^ This discrepancy could be due to variations in lifestyle, different criteria used to diagnose hypertension and differences in the duration and severity of diabetes.

Although hypertension is easily diagnosable and treatable with lifestyle modifications, its burden has been increasing in Ethiopia, with a high rate of hospitalization and mortality.^20^ However, controlling DM provides an entry point for dealing with hypertension and other non-communicable diseases. To alleviate the burden of hypertension associated with DM, a holistic approach to patient care, including an early diagnostic approach, should be the strategy in Ethiopia.^20^

## Strengths and limitations

As a strength, this systematic review and meta-analysis were the first study that addressed the pooled prevalence of hypertension among type 2 DM patients in Ethiopia, and several databases were used to search articles. As a limitation, interpretation of the data needs to be done with caution, as the heterogeneity (interstudy variability) was very high given the use of random effects models to accommodate the variability. A small number of studies were used in this study, and this prevented us from stratifying the analysis of time trends by the quality of the research. Additionally, this meta-analysis was also limited in evaluating the pooled factors causing hypertension.

## Conclusions and recommendations

This study showed a high prevalence of hypertension among type 2 DM patients in Ethiopia. This evidence indicates that double-burden diseases affect Ethiopia, so policymakers and other relevant agencies should give attention and priority to reducing hypertension and improving the standard of care for DM patients in Ethiopia. Condition-based approaches and country context-specific appropriate preventive measures should be implemented to reduce the burden of hypertension among DM patients and increase the overall quality of healthcare services. A comprehensive and multifaceted approach is needed to counter the complications associated with diabetes, focusing on adherence to treatment, comorbidity management, screening and early diagnosis of a condition. Additionally, further studies are required to determine predisposing factors for hypertension development among patients with DM.

## Supplementary Material

ihac060_Supplemental_Files

## Data Availability

All raw data generated or analysed during the current study are available from the corresponding author upon a reasonable request.
