# Supplementary material for: Prevalence of hypertension among type 2 diabetes mellitus patients in Ethiopia: a systematic review and meta-analysis
Source: Int Health. 2022 Sep 2;15(3):235–41. doi: 10.1093/inthealth/ihac060 (PMC10153558; doi:10.1093/inthealth/ihac060)
Supplement: ihac060_Supplemental_Files [file ihac060_supplemental_files.zip › Additional file 3.docx]

***Additional file 3: Studies search strategies and entry terms from different electronic databases on the Prevalence of Hypertension among Type 2 Diabetes Mellitus Patients***

**Sample search string for Medline database, EBSCO host Interface**

| **#** | **Query** | **Limiters/Expanders** | **Last Run Via** | **Results** |
| --- | --- | --- | --- | --- |
| S4 | (Ethiopia) AND (S1 AND S2 AND S3) | Search modes - Find all my search terms | Interface - EBSCOhost Research Databases  Search Screen - Advanced Search  Database - MEDLINE | 23 |
| S3 | Ethiopia | Search modes - Find all my search terms | Interface - EBSCOhost Research Databases  Search Screen - Advanced Search  Database - MEDLINE | 9097 |
| S2 | Hypertension OR High blood pressure OR Diastolic OR systolic blood pressure OR Diabetic Mellitus | Search modes - Find all my search terms | Interface - EBSCOhost Research Databases  Search Screen - Advanced Search  Database - MEDLINE | 432 |
| S1 | Prevalence OR magnitude | Search modes - Find all my search terms | Interface - EBSCOhost Research Databases  Search Screen - Advanced Search  Database - MEDLINE | 1234 |

| **Google scholar database** | |
| --- | --- |
| Ethiopia Hypertension OR "Systolic Blood Pressure, High " OR " Diastolic Blood Pressures, High" OR "prevalence" OR "magnitude" AND "Diabetic Mellitus" | 38 |
| **Hinari database** | |
| ((TitleCombined:(Hypertension)) OR (TitleCombined:("Systolic Blood Pressure, High")) OR (TitleCombined:("Diastolic Blood Pressures, High")) OR (TitleCombined:("Prevalence of Hypertension")) OR (TitleCombined:("Diabetic Mellitus"))) AND (TitleCombined:(Ethiopia)) | 42 |
| ((Abstract:(Hypertension)) OR (Abstract:("Diastolic Blood Pressure, High")) OR (Abstract:("Systolic Blood pressures, High")) OR (Abstract:("High Blood Pressure")) OR (Abstract:("Diabetic Mellitus"))) AND (Abstract:(Ethiopia)) | 115 |
