# Supplementary material for: Prevalence of hypertension among type 2 diabetes mellitus patients in Ethiopia: a systematic review and meta-analysis
Source: Int Health. 2022 Sep 2;15(3):235–41. doi: 10.1093/inthealth/ihac060 (PMC10153558; doi:10.1093/inthealth/ihac060)
Supplement: ihac060_Supplemental_Files [file ihac060_supplemental_files.zip › Additional file 2.docx]

**Appraisal**

Table 1: Critical appraisal check list of quantitative studies of prevalence of hypertension among diabetic patients in Ethiopia (1 =yes, 0=no/not mentioned); total score=8

| Studies | Q1 | Q2 | Q3 | Q4 | Q5 | Q6 | Q7 | Q8 | Total score | Remark |
| --- | --- | --- | --- | --- | --- | --- | --- | --- | --- | --- |
| Dedefo et al. | Y | Y | Y | Y | Y | Y | Y | Y | 8/8 |  |
| Tadesse et al. | Y | Y | Y | Y | Y | U | Y | Y | 7/8 |  |
| Muleta et al. | Y | Y | Y | Y | Y | N | Y | Y | 7/8 |  |
| Kehabtimer, S Kotiso., et al. | Y | Y | Y | Y | Y | Y | Y | Y | 8/8 |  |
| Dagnew and Yeshaw | Y | Y | Y | Y | Y | Y | Y | Y | 8/8 |  |
| Akalu Y. and Belsti Y. | Y | Y | Y | Y | Y | Y | Y | Y | 8/8 |  |

Notes:

Q1 - Were the criteria for inclusion in the sample clearly defined?

Q2 - Were the study subjects and the setting described in detail?

Q3 - Was the exposure measured in a valid and reliable way?

Q4 - Were objective, standard criteria used for measurement of the condition?

Q5 - Were confounding factors identified?

Q6 - Were strategies to deal with confounding factors stated?

Q7 - Were the outcomes measured in a valid and reliable way?

Q8 - Was appropriate statistical analysis used?

Abbreviations: Y, yes; N, no; U, unclear.
